# Posttraumatic hydrocephalus as a complication of decompressive craniectomy–same old story, new perspectives

**DOI:** 10.3389/fsurg.2024.1415938

**Published:** 2024-08-07

**Authors:** Nicoleta-Larisa Șerban, Ioan Stefan Florian, Ioan Alexandru Florian, Andreea Atena Zaha, Daniela Ionescu

**Affiliations:** ^1^ Clinic of Neurosurgery, Cluj County Emergency Clinical Hospital, Cluj-Napoca, Romania; ^2^ Department of Neurosurgery, “Iuliu Hatieganu” University of Medicine and Pharmacy, Cluj-Napoca, Romania; ^3^ Faculty of Medicine, “Iuliu Hațieganu” University of Medicine and Pharmacy, Cluj-Napoca, Romania; ^4^ Department of Anesthesia and Intensive Care I, “Iuliu Haţieganu” University of Medicine and Pharmacy, Cluj-Napoca, Romania; ^5^ Association for Research in Anesthesia and Intensive Care (ACATI), Cluj-Napoca, Romania; ^6^ Onco-Anaesthesia Research Group, ESAIC, Brussels, Belgium; ^7^ Outcome Research Consortium, Cleveland, OH, United States

**Keywords:** decompressive craniectomy, complications, posttraumatic hydrocephalus, mechanical ventilation, vasopressors

## Abstract

**Objective:**

Decompressive craniectomy (DC) serves as a vital life-saving intervention, demonstrating efficacy in reducing intracranial pressure (ICP). However, its efficacy hinges on meticulous surgical execution, perioperative management, and vigilance toward potential complications. The incidence of complications associated with DC plays a pivotal role in determining its superiority over medical management for patients experiencing intracranial hypertension following traumatic brain injury (TBI).

**Methods:**

Severe cases often require more intensive therapy, prolonged mechanical ventilation, and vasopressor treatment. Identifying the optimal moment for early extubation and minimizing vasopressor use is crucial to reducing the risk of complications, including PTH. Our study aims to highlight the potential risks associated with prolonged mechanical ventilation and long-term vasopressor administration. The collected data were demographics, the craniectomy size, the distance from the midline of the craniectomy, the presence or absence of hydrocephalus, duration of mechanical ventilation and vasopressor treatment, and outcome at 30 days.

**Results:**

Seventy-two patients with a mean age of 44.2 (range 5–83) were included in the study, with a median craniectomy size of 119.3 cm^2^. In our series, craniectomy areas ranged between 30 and 207.5 cm^2^ and had a similar decrease in midline shift in all cases. We did not observe any associations between the surface of craniectomy and the complication rate (*p* = 0.6302). There was no association between craniectomy size and mortality rate or length of hospital stay. The most common complication of decompressive craniectomy in our study group was posttraumatic hydrocephalus, with an incidence of 13.8%. Our results showed that craniectomy size did not independently affect PTH development (*p* = 0.5125). Still, there was a strong correlation between prolonged time of vasopressor treatment (*p* = 0.01843), period of mechanical ventilation (*p* = 0.04928), and the development of PTH.

**Conclusions:**

This study suggests that there is no clear correlation between craniectomy size, midline shift reduction, and survival rate. An extended period of vasopressor treatment or mechanical ventilation is linked with the development of posttraumatic hydrocephalus. Further studies on larger series or randomized controlled studies are needed to better define this correlation.

## Introduction

In contrast to other organ systems, the central nervous system is placed in a closed, rigid compartment (1). Consequently, even slight alterations in intercompartmental volume can induce significant fluctuations in intracranial pressure (ICP).

Clinical therapy for traumatic brain injury (TBI) is still inadequate despite its status as a prevalent cause of permanent disability (2). Untreated TBI frequently results in elevated ICP, which can be fatal or cause permanent disability. Clinicians frequently utilize stepwise acute care strategies to decrease intracranial pressure (ICP). However, certain patients may not show improvement with medicinal treatment and may be deemed suitable for surgical decompression.

Over several decades, decompressive craniectomy (DC) has become a crucial surgery for managing neurosurgical emergencies. Decompressive craniectomy is not only one of the oldest procedures performed in cranial neurosurgery but also one of the most commonly performed during neurosurgical training. This highlights its significance in both clinical practice and education for neurosurgeons in training (3). It effectively reduces intracranial pressure, thereby saving lives (2). Despite the straightforward nature of the procedure, the exact place of DC in the management of various neurologic injuries, such as TBI, needs to be better established, mainly because, in some instances, the procedure may be even harmful (2).

The justification for its utilization is grounded in the Monro-Kellie hypothesis, with the first description of the process provided by Kocher in 1901 and Cushing in 1905 (4), being ever since a matter of much debate.

Following a traumatic brain injury, the use of decompressive craniectomy can result in a range of therapeutic outcomes. These include the expansion of the intracranial space and intracranial volume, leading to the restoration of peri mesencephalic cisterns. Additionally, decompressive craniectomy can reduce midline displacement, enhance cerebral compliance (5), reduce intracranial pressure (ICP) (27), elevate cerebral blood flow and perfusion (5), and improve cerebrovascular regulation (7). Despite these favorable effects, numerous disputes persist with respect to specific facets of the surgical procedure, encompassing the most suitable patient demographics, time, and method. There are multiple technical variations of this procedure, and without clear evidence of superiority among these variations, their discussion is beyond the scope of the study.

Determining the ideal extent of craniectomy, which achieves maximal decompression while minimizing complications, remains uncertain. Some suggest a craniectomy diameter of 12 cm as the minimum size for effective decompression, as smaller defects correlate with a notable rise in hemicraniectomy-associated lesions (8). It is not yet routine practice, but it might be more useful for comparing the effectiveness of craniectomy if the decompressed area is reported as a percentage of the total surface area of the hemicranium. In this way, an area of 100 cm^2^ would represent 50%, 60%, or 70% of the hemicranial surface, depending on the size of the skull, and the decompressive effect could be evaluated more realistically. However, this will be the subject of a future study. Posttraumatic hydrocephalus (PTH) stands out as a frequent complication of decompressive craniectomy (DC), yet our understanding of its development relies mainly on suppositions and hypotheses rather than empirical evidence.

In this regard, considering that respiration and especially inspiration is a major regulator of CSF flow (9), the arising question is whether the period of mechanical ventilation is correlated with the development of PTH. Mechanical ventilation, although primarily intended to improve brain oxygenation, is not a physiological process, and the inspiration-expiration phases of medical equipment cannot mimic physiological breathing in all its components. Therefore, CSF circulation is at least altered under its respiratory component influence. Moreover, the presence of the patient's spontaneous breathing (lightening sedation, lightening coma, awakening, coughing due to mechanical irritation) interfering with the equipment creates moments of increased intrathoracic and intracranial pressure, further altering fluid circulation.

To date, we are not aware of any real-time MRI study regarding CSF kinetics under mechanical ventilation conditions, but such a study could provide additional elements concerning the influence of mechanical ventilation on the development of PTH.

These are just a few elements that come together to complete a picture that is still not fully understood regarding the development of PTH. Far from being a mechanical phenomenon of simple water accumulation in the ventricular system, PTH is the result of complex mechanisms in which the roles of malresorption at the areas of encephalomalacia, transmantle pressure gradient, osmotic gradient, pulsatility aberration, vascular bed dysfunction, aquaporin channels alterations, reduced brain compliance, and brain distortion will need to be understood one by one.

As a consequence, alterations of brain pulsatility already compromised by DC will impede the CSF flow and possibly influence PTH development (10).

In a lack of evidence for optimal craniectomy size, the current study investigates whether the size of craniectomy correlates with the risk of developing complications. We establish a secondary objective: the possible correlations in between the duration of mechanical ventilation, vasopressor treatment and development of PTH.

## Materials and methods

This retrospective study was approved by the Institutional Ethics Committee of Cluj County Emergency Hospital (No. 1032/61/13.01.2021).

We retrospectively reviewed all patients undergoing decompressive craniectomy from January 2019 to December 2023 in the Department of Neurosurgery, Cluj County Clinical Emergency Hospital, as documented in our electronical patient data base.

Based on the inclusion criteria, all patients who underwent a decompressive craniectomy with a primary diagnosis of traumatic brain injury were included. Part of the technique for additional reduction of intracranial pressure consists of opening the dura mater in a star-shaped manner, starting with an arcuate line over the Sylvian fissure, completed with additional radial incisions along the main drainage veins is the standard practice in our department. Closure of the dura mater is achieved with autologous periosteum anchored loosely, so that the entire surface of the decompressed area is covered.

Exclusion criteria encompassed patients who underwent craniectomy for alternative reasons such as malignant middle cerebral artery (MCA) infarction, aneurysmal subarachnoid hemorrhage (SAH), or tumors. Additionally, patients without pre-operative imaging within 6 h of surgery or post-operative imaging within 7 days were also excluded from the study.

Intracranial pressure monitoring could only be systematically performed in some patients due to a small number of electronic intracranial pressure monitors available and discontinuity of intraparenchymal electrode supply. Alternatively, in a proportion of cases, intraventricular pressure measurement was performed at the same time as the placement of the external ventricular catheter. However, this was performed in a small number of cases due to the fact that the size of the ventricles did not allow safe placement of the ventricular catheter without an increased risk of additional parenchymal damage. Therefore, direct measurement of intracranial pressure was not part of the criteria applicable to the present study.

The utilization of postoperative imaging facilitated the determination of the extent of shift or effacement resolution. The on-call consultant neurosurgeon responsible for the patient's care made the decision to perform a decompressive craniectomy, taking into consideration the patient's demographics, clinical condition, imaging results, and history of neurotrauma.

For all patients, clinical and demographic characteristics such as age, sex, GCS at admission, and pupils' symmetry were recorded. Outcome measures included length of hospital/intensive care unit stay, length of mechanical ventilation, and vasopressor treatment. Furthermore, a comprehensive assessment of all accessible serial CT scans from admission to discharge was conducted to identify convexity and interhemispheric subdural hematomas intraparenchymal hematomas or hemorrhagic contusions (HC), intraventricular hemorrhage, and hydrocephalus. The midline shift length on CT, the size of the craniectomy, and the distance of the craniectomy from the midline were also measured. Patients were monitored dynamically, typically with an initial CT evaluation followed by re-evaluation every 8–12 h depending on the patient's clinical condition. Immediately after performing the craniectomy, we routinely conduct a CT scan primarily for medical-legal purposes (to demonstrate the evacuation of the hematoma, removal of bone fragments, or the immediate postoperative imaging of the brain). A follow-up evaluation at 24 h postoperatively is part of our standard protocol, and subsequent CT scans are performed based on the patient's clinical condition.

**Figure 1:**
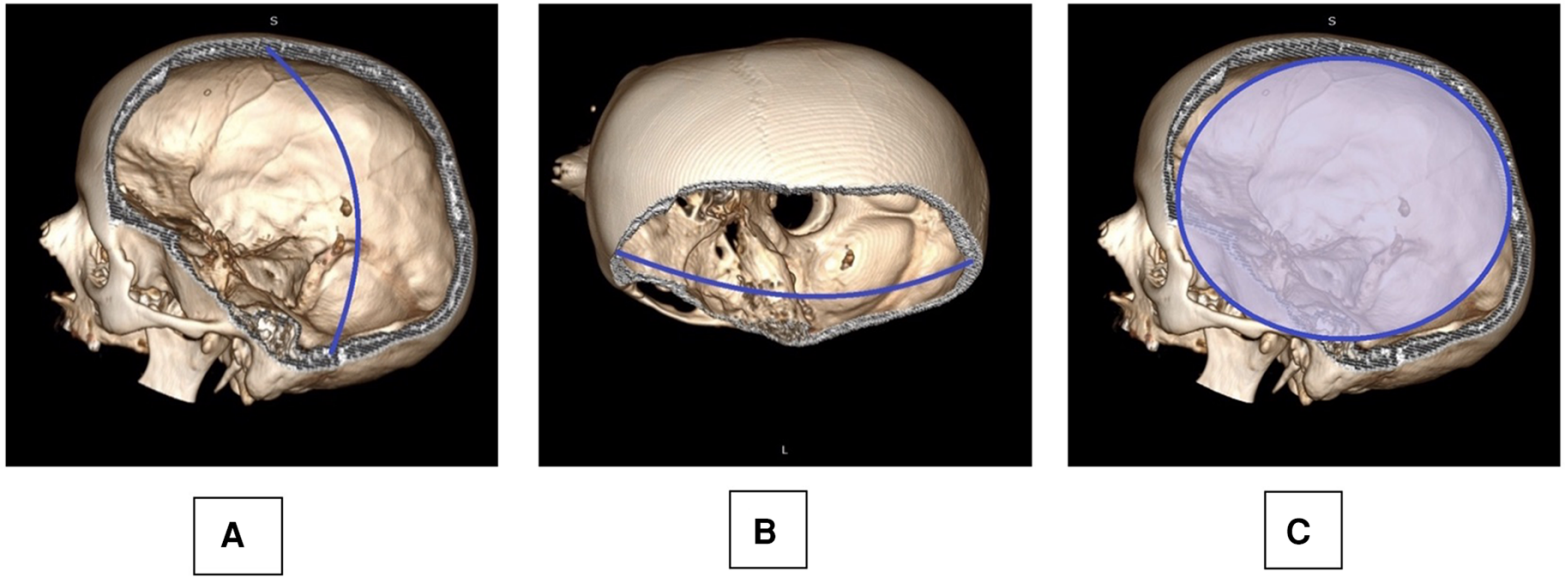
Skull reconstruction based on a postoperative CT scan is provided: the maximum cranio-caudal (**A**: CC) and anterior-posterior (**B**: AP) diameter were measured, taking into account also the anatomical curvature of the skull as observed on the contralateral side. A surface estimate of the decompression (**C**: SE) was deducted from the AP and CC measurements: SE=AP/2×CC/2×π.

Early postoperative CT scans were analyzed using an open-source DICOM viewer, and the extent of bony decompression was calculated as follows: Maximum anterior-posterior and maximum craniocaudal (CC) diameter, surface estimate (SE) of bony decompression (SE = AP/2 × CC/2 × π) (11) (Figure 1).

The primary radiological indicators for performing DC were midline shift over 5 mm, disappearance of basal cisterns, and mass effect of cerebral lesions. Most cases involved acute subdural hematoma, traumatic intraparenchymal hemorrhage, or multifocal post-traumatic cerebral contusion. Specific radiological features, such as subarachnoid hemorrhage (SAH), were present in all cases and could influence the occurrence of PTH. A midline is a line that connects the frontal bone to the occipital bone, starting with the anterior connection of the falx cerebri. The midline determination and shift measurement were conducted on a single axial CT slice, specifically at the rostrocaudal midpoint of the lateral ventricle.

The distance of craniectomy from the midline was calculated through the analysis of 3D reconstructions from CT scan images.

The cerebral lesions detected through computed tomography were further categorized based on the Marshall scoring system.

Posttraumatic hydrocephalus (PTH) was a primary focus of our study. Acute hydrocephalus cases were not included. We defined PTH as the development of ventriculomegaly at a variable postoperative interval, characterized by the progressive enlargement of the ventricles, the presence of transependymal edema, concurrent brain herniation at the craniectomy site, and the deterioration of neurological status following an initial period of improvement.

In all patients, the duration of mechanical ventilation and the duration and mean dose of vasopressor were also recorded.

Statistical analysis was done with SPSS 18.0. Categorical variables were summarized using frequencies and percentages, while continuous variables were presented as means with standard deviations (mean ± SD). Two-variance comparisons were performed using the *F*-test and Welch Two Sample Test. Logistic regression analysis was utilized to assess the association between these characteristics and craniectomy size. A significance level of *P* < 0.05 was considered statistically significant. To account for the interaction between multiple variables, we included a multivariate logistic regression analysis. This method allows us to control for multiple confounding variables simultaneously and provides adjusted Odds Ratios (OR) and Confidence Intervals (CI) for each factor, offering a more precise understanding of their relationship with PTH. We included variables such as age, sex, duration of mechanical ventilation, duration of vasopressor treatment, and craniectomy size. Patients were categorized based on the presence or absence of PTH. We used a logistic regression model to estimate the probability of developing PTH, adjusting for potential confounders. The model is specified as follows:

$$\begin{array}{rl} & ‘‘logit(pth)\;=\;\beta 0\;+\;\beta 1\;\times \;age\;+\;\beta 2\;\times \;sex\;+\;\beta 3\\ & \times \;duration\_ventilation\;+\beta 4\times duration\_vasopressor+\beta 5\\ & \times craniectomy\_sizelogit(pth)=\beta 0+\beta 1\;\times \;age+\beta 2\;\times \;sex+\beta 3\\ & \times \;duration\_ventilation+\beta 4\;\times \;duration\_vasopressor+\beta 5\\ & \times \;craniectomy\_size" \end{array}$$

## Results

Out of 107 patients undergoing DHC within the given period, we included 72 patients based on the availability of comprehensive clinical data sets and postoperative CT scans. Thirty-five patients were excluded from this study for incomplete clinical and diagnostic datasets. The age distribution of the patients in our study was as follows: 5 patients were under 18 years old, 13 patients were between 18 and 30 years old, 34 patients were between 30 and 60 years old, 18 patients were between 60 and 75 years old, and 2 patients were over 75 years old. All patients had an associated mass lesion. In our study, 59 out of the 72 patients (approximately 81.9%) were presented with acute subdural hematoma (HSDAc), highlighting the prevalence of this condition among the cases analyzed. Admission GCS ranged from 3 to 8, with a mean of 3.44. Pre-operative pupil response was normal in 41.7% of cases, unilaterally fixed in 31.9%, and bilaterally fixed and dilated in 26.4%. The most common mechanism of trauma was falls in 32 patients, followed by 21 traffic accidents, three aggressions, and 16 other causes.

In our study, the complication rates were analyzed, revealing that 5 patients rebleed and 3 of them need further surgeries, also 8 patients developed infection-related complications, and 2 of these were cases of meningitis.

The initial computed tomography (CT) findings revealed the presence of a mass lesion accompanied by cerebral edema or mass effect in all cases. Craniectomy was performed as the first measure in 67 cases, while only 5 cases underwent decompressive craniectomy as a last-resort treatment. The size of the craniectomy was determined by the operating surgeon's judgment in all instances.

**Figure 2:**
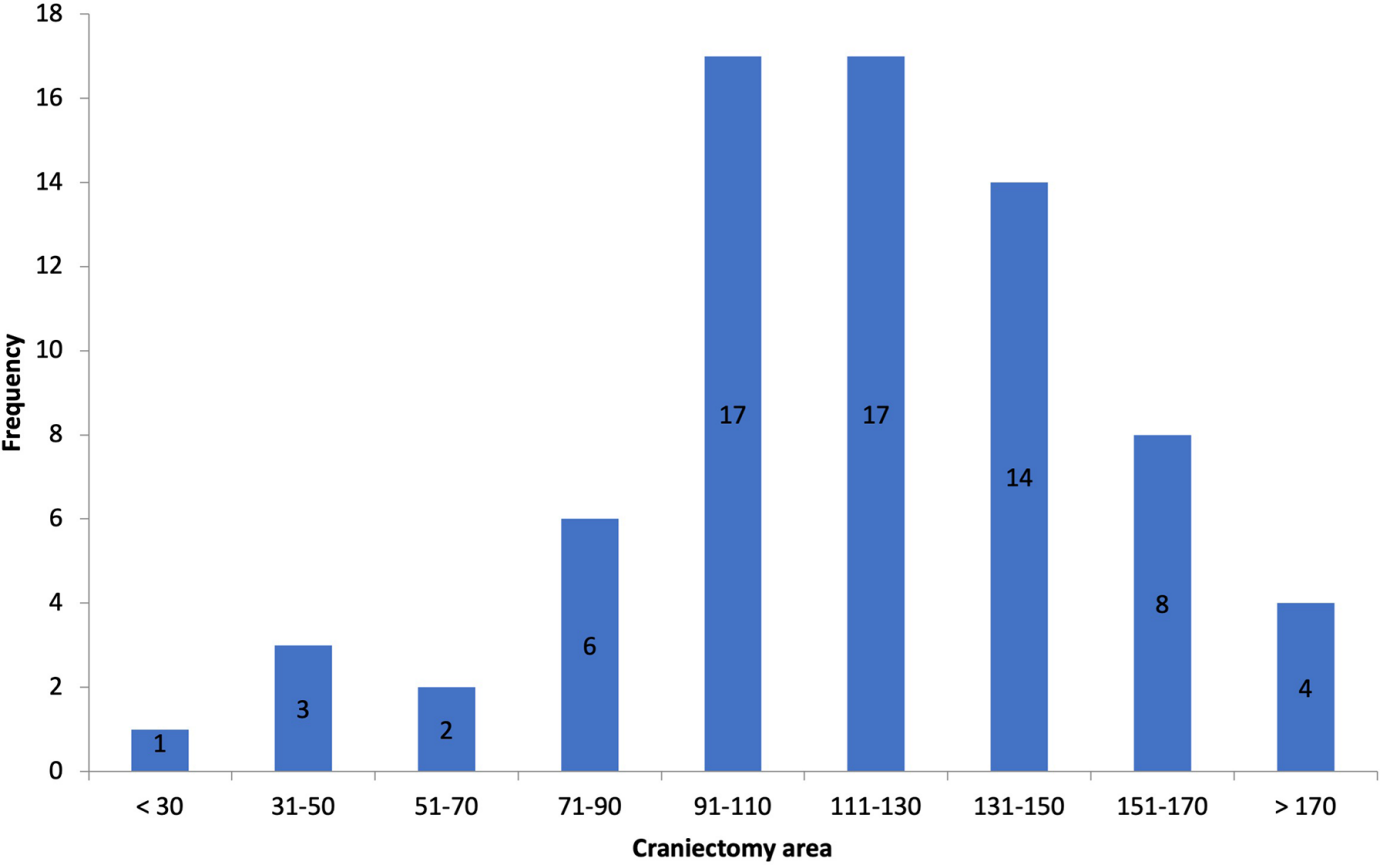
Histogram of craniectomy size.

Postoperative scans showed an average craniectomy diameter of 13.4 ± 1.2 cm (AP, range 8.78–23 cm) and 11.6 ± 0.9 cm (CC, range 6.8–14.8 cm), respectively, with an average SE of 119.3 cm^2^ (range 30–207.5 cm^2^), defining our thresholds for categorization (Figure 2). The European Brain Injury Consortium indicated that a craniectomy size of less than 30 cm^2^ was insufficient, and obtained an average craniectomy size of 67 cm^2^ (12). Based on this consideration, all the bone flaps in our research were of adequate size.

**Figure 3:**
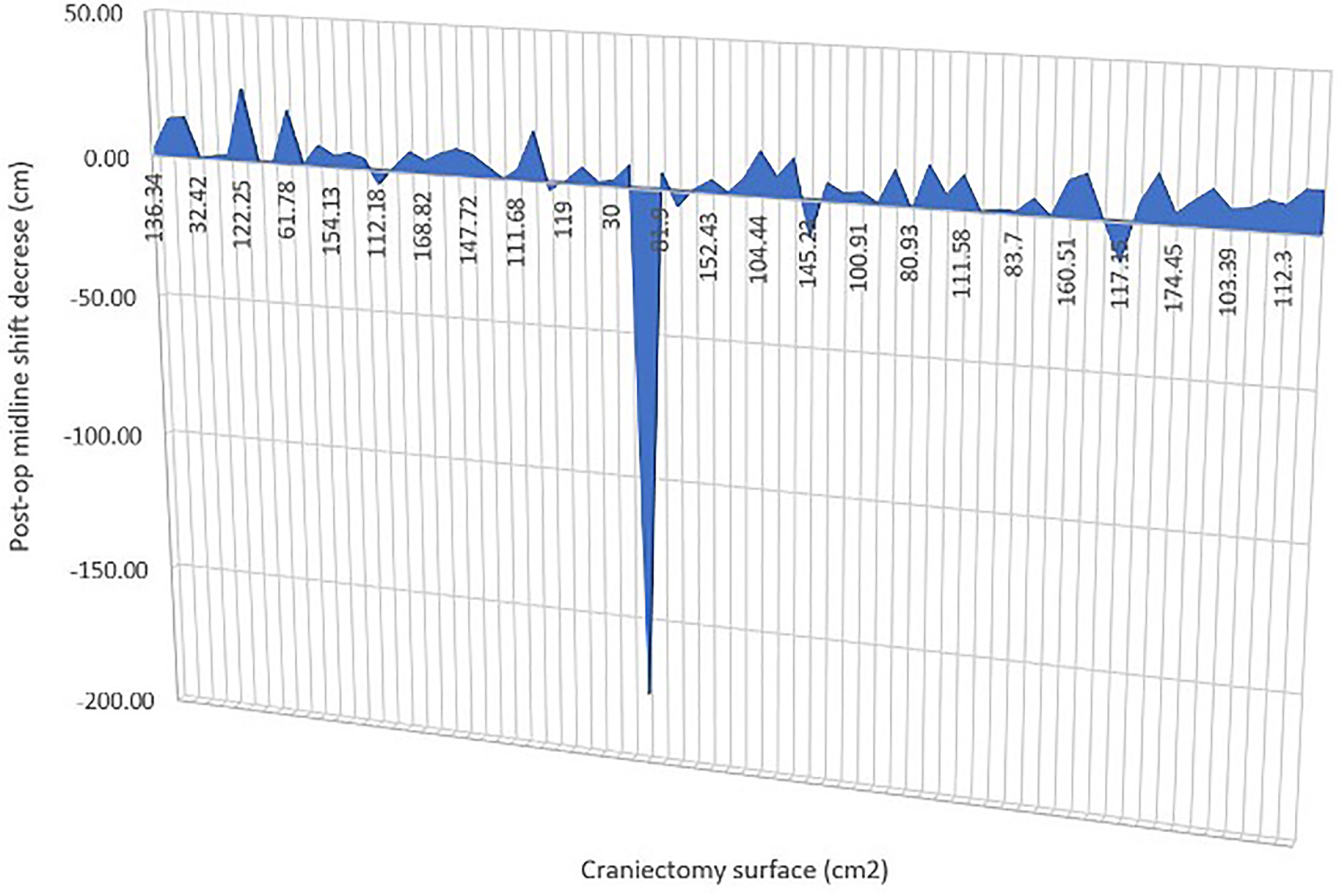
Graph to show the change in midline shift in relation to craniectomy.

In our series, we had craniectomy areas ranging between 30 (bifrontal craniectomy in a child) and 207.5 cm^2^ and had similar decrease in midline shift across all of the cases, with one exception that is not statistically relevant (*p*-value = 0.3362) (Figure 3).

The median craniectomy area correlated with mortality was 129.93 cm^2^; however, there was no association between the mortality rate and the craniectomy area (*p* = 0.7322) in our study.

There was no significant correlation between the extent of the craniectomy and, the rate of each complication and the overall complication rate. Twelve patients experienced complications after surgery. The most common complication of decompressive craniectomy in our data set was PTH, with an incidence of 13.8%.

**Figure 4:**
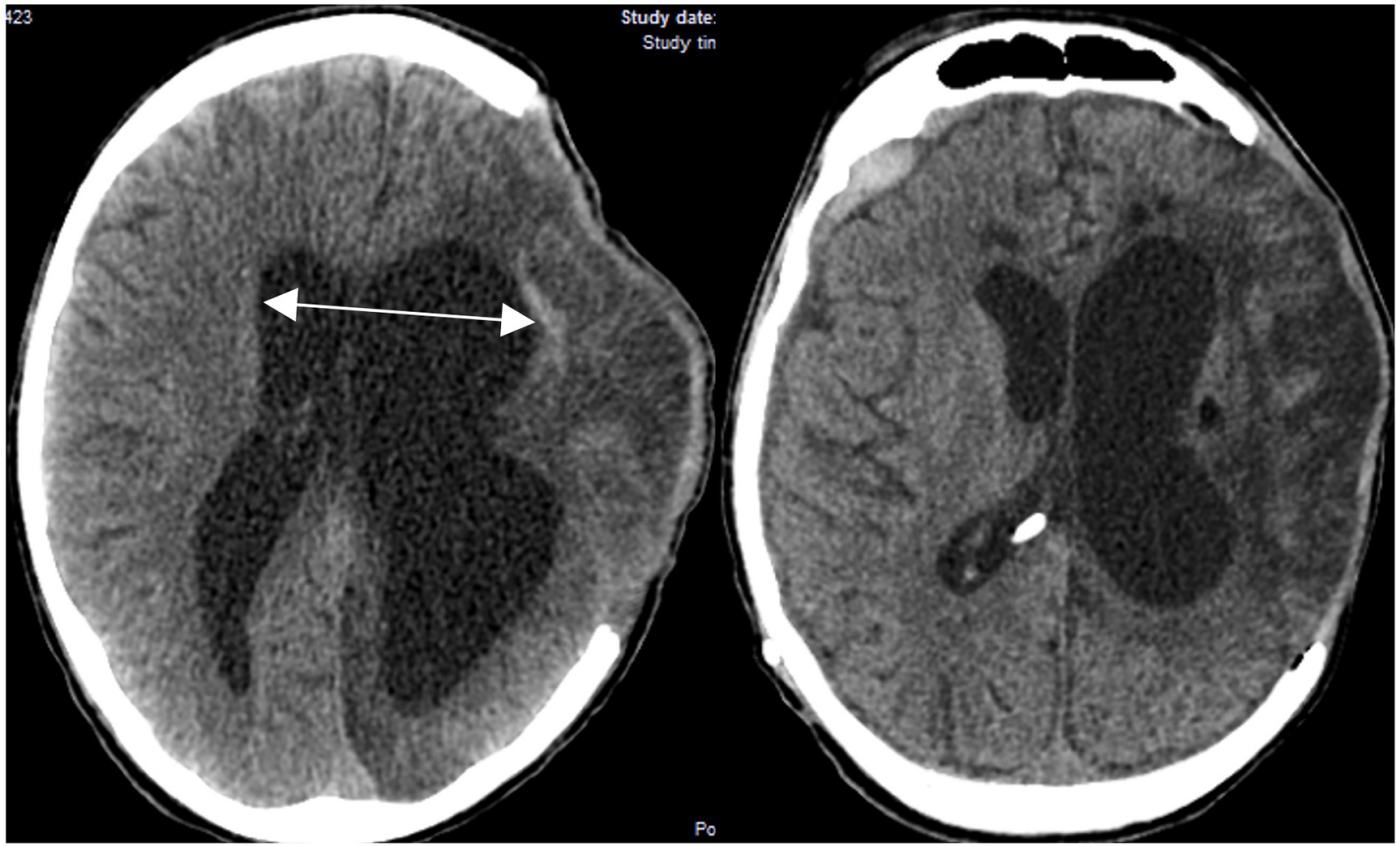
CT brain showing posttraumatic hydrocephalus after decompressive craniectomy.

Indications of ventricular dilatation and trans ependymal edema on CT, clinical deterioration or lack of improvement over time, and clinical improvements observed after ventriculoperitoneal shunting were utilized to determine the presence of hydrocephalus (Figure 4).

The mean area of the bone flap in the hydrocephalus group was 120.53 cm^2^, which is not significantly statistically different from that of the non-hydrocephalus group, where the mean craniectomy area was 116.41 cm^2^ (*p*-value = 0.5125).

**Figure 5:**
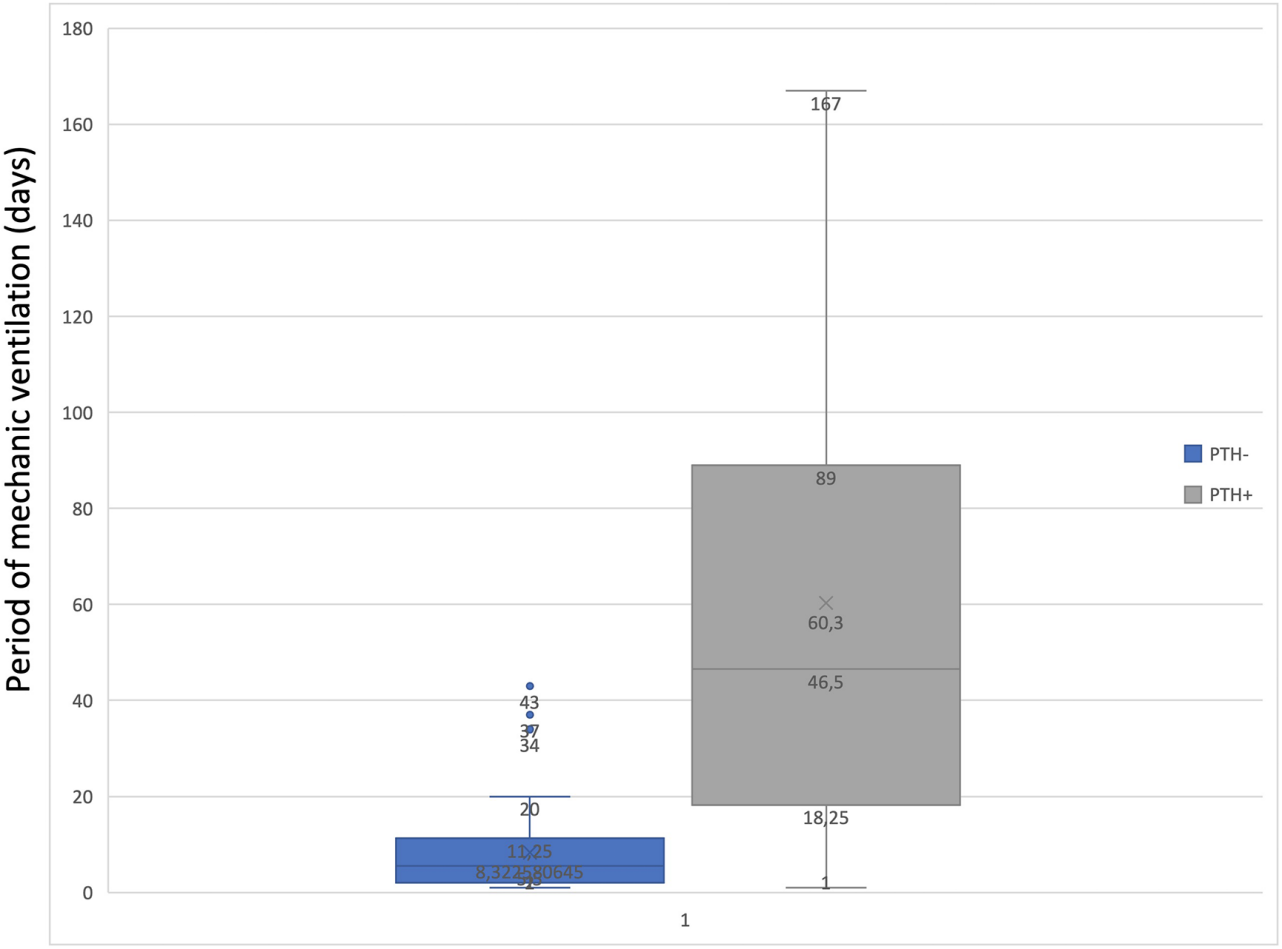
Graph to show the correlation between the period of mechanic ventilation and PTH.

**Figure 6:**
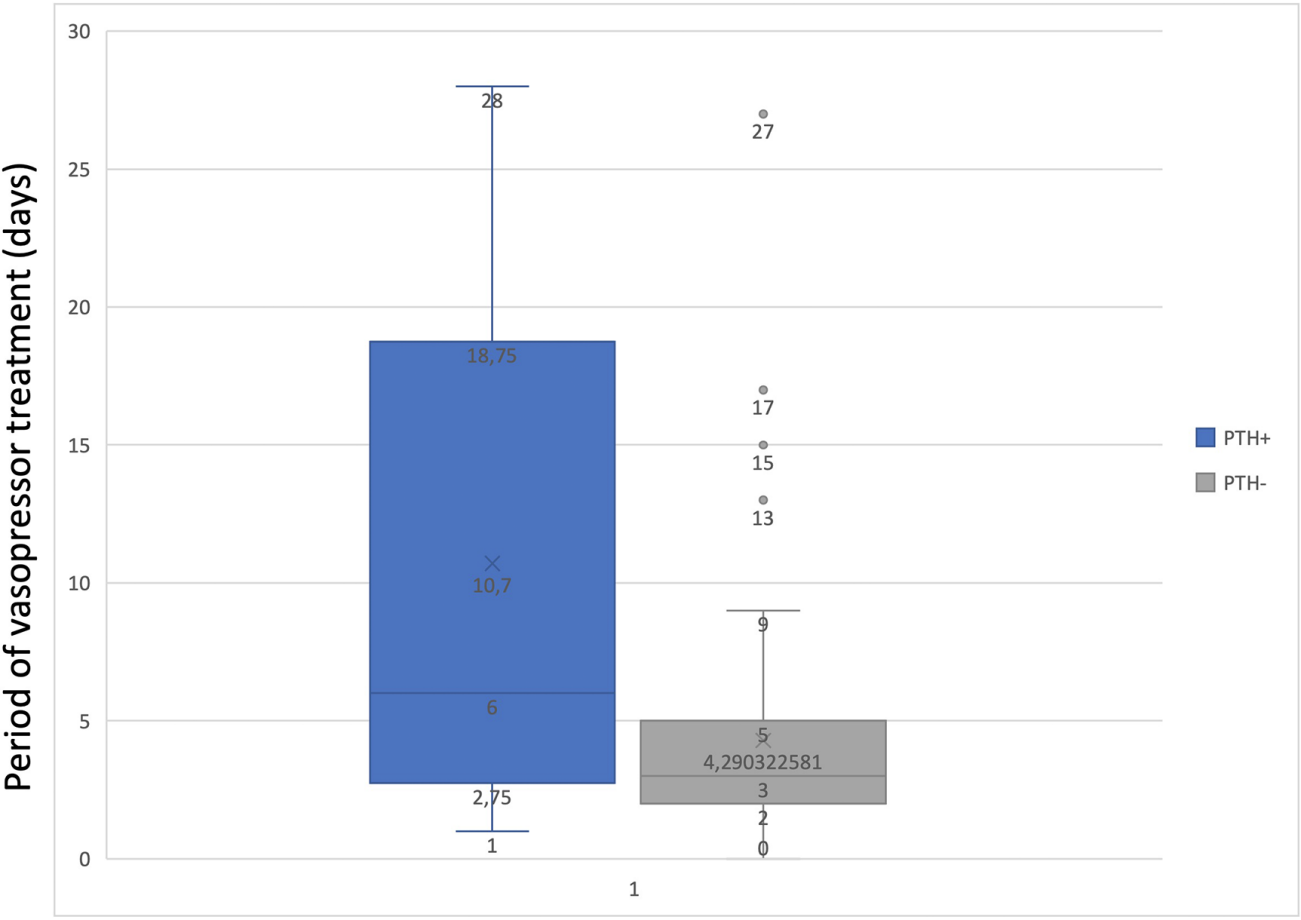
Graph to show the correlation between the period of vasopressor treatment and PTH.

To determine the statistical relationship between posttraumatic hydrocephalus and length of mechanical ventilation or vasopressor treatment, we investigated whether correlations were present. There was a statistically significant correlation (*p* = 0.01843) between long-term vasopressor treatment and the occurrence of PTH (Figure 5). Furthermore, an increased mechanical ventilation period in patients who developed PTH was discovered (*p* = 0.04928) (Figure 6).

Early tracheostomy is known to potentially reduce the need for prolonged sedation and associated complications in critically ill patients. Despite its benefits, in our study, only one out of the 72 patients underwent early tracheostomy, which could result in a reduction of sedative medication.

Of the ten patients who developed hydrocephalus, seven patients (70%) had undergone craniotomy whose superior limit was <25 mm from the midline.

## Discussion

Although a decompressive craniectomy is one of the oldest and most frequently performed cranial neurosurgery procedures, the precise reasons and reasoning for it are still being clarified (5).

Several studies have focused on bringing some light on the optimal craniectomy size. In 2008, Gao et al. l attempted to use biomechanical modeling to determine an optimal craniectomy size (13). In the multivariate analysis of 526 cases of DC for many neurosurgical conditions, including traumatic brain injury, the size of the bone flap greater than 12 cm^2^ was an independent predictor factor for survival in patients younger than 65 years and older than 18 years (14).

Jiang and Qiu compared the influence of a larger fronto-temporo-parietal bone flap vs. a smaller DC. They found more significant mortality in the smaller DC group and better neurological outcomes in the larger DC group (15).

Our examination did not uncover any notable correlation between the extent of the craniectomy and patient outcomes or complication rates. Nevertheless, it is essential to acknowledge that our study had a smaller sample size compared to prior research. Nonetheless, our findings regarding mortality rates, outcomes, and complication rates align with previously published data—similar to our results, Sedney et al. (16) also observed no increase in complications associated with larger bone flap sizes. However, we did not observe any differences in other outcome measures, such as the duration of hospital or ICU stays, in our study. Additionally, factors like the initial severity of the injury are likely to influence the ultimate outcome for patients.

Recently, Harris et al. in 2020 demonstrated that the dimensions of the craniectomy had no correlation with the amount of midline shift (17). This can reasonably be explained by the argument that residual hematoma, brain swelling, and underlying parenchymal injury are the primary factors contributing to midline shift. Consistent with the results of Harris' study, our investigation did not identify any significant reduction in midline shift that could be linked to craniectomy size.

Nevertheless, a larger craniectomy size has been linked to higher rates of complications, and DC is acknowledged as a surgery with significant morbidity, with an overall complication incidence of 55% reported in a retrospective analysis of a large cohort of patients undergoing this procedure (18).

A limited number of studies proposed that a larger craniectomy size might pose a risk for hydrocephalus development due to potential interference with hydrodynamic cerebrospinal fluid (CSF) circulation. Thus, a larger craniectomy area can serve as a significant independent risk factor for post-decompressive craniectomy hydrocephalus (18). According to our results, the craniectomy size did not affect the development of PTH independently. The size of the decompressive craniectomy is not only thought to be related to post-traumatic hydrocephalus but also to the syndrome of the trephined, an underestimated and poorly understood complication. This syndrome can significantly impact patient recovery and outcomes, emphasizing the need for careful consideration of craniectomy size during surgery (19).

To see what is/if there is a contribution of mechanical ventilation in the development of hydrocephalus in severely injured patients, previous studies reported that a longer duration of unconsciousness was associated with a higher risk of developing hydrocephalus after TBI (20, 21).

While regular breathing triggers strong but fluctuating cerebrospinal fluid (CSF) flow, the notable changes observed during forced respiration indicate that the decrease in thoracic pressure during inhalation serves as the underlying mechanism. These findings align with previous reports of differing CSF flux in the spinal canal during regular vs. forced breathing conditions, as detected by an early one-dimensional MRI technique (22). The same principle extends to the motions of tagged brain CSF MRI signals, as recently documented by Yamada et al. in their comparison of extended periods of inhalation and exhalation (23). Episodes of sedation interruption, whether unintended or deliberate for periodic neurological assessments, can lead to instances of coughing, Valsalva maneuver, or even patient resistance against mechanical ventilation. It was demonstrated that these events result in sudden morphological alterations in the adjacent structures near the tentorial hiatus and craniectomy edges, potentially impacting brain function and cerebrospinal fluid (CSF) dynamics (24).

Our study found that patients who developed PTH were mechanically ventilated for a mean time interval of 60 days, compared with those who did not develop these complications with a mean of 11 days of mechanical ventilation. This correlation could indicate that mechanical ventilation plays a role in developing PTH, but this study was not designed to prove causality. Further studies should focus on more extensive series to assess this.

The literature offers limited evidence on how vasopressors will increase the pulsatility of the brain with consequences in the CSF (patho)physiology. Our case series was able to provide a correlation between long-term vasopressor treatment and the occurrence of PTH. Still, the current literature does not provide relevant data to support this finding. We can only hypothesize that vasopressors might impact brain pulsatility, thereby altering the pulsatile pressure gradient between the ventricle and an already compromised subarachnoid space, in line with Greitz's Hydrodynamic theory of hydrocephalus development (10). Not to be neglected is the fact that prolonged use of vasopressors, particularly Norepinephrine, may induce vasoconstriction in major vessels, potentially resulting in ischemic lesions. These lesions, combined with primary and secondary traumatic brain injury (TBI), could contribute to the onset of encephalomalacia (25). To the best of our knowledge, this is the first study that establishes a significant correlation between the duration of vasopressor and PTH development in patients with DC.

Recently, Waziri and colleagues (18) suggested that decompressive craniectomy might contribute to the alteration of the typically dicrotic cerebrospinal fluid (CSF) pulse waveform observed in patients undergoing this procedure. Due to the pressure-dependent one-way valves of the arachnoid granulations, which connect the subarachnoid space to the draining venous sinuses, it is likely that opening the cranial vault can disrupt the pulsatile intracranial pressure dynamics, leading to a decrease in cerebrospinal fluid (CSF) outflow. According to Aline et al., removing the skull in close proximity to the midline decreases the external strain exerted on the veins, particularly during the diastolic period. This alteration leads to an increase in venous outflow, resulting in increased absorption of extracellular fluid and a subsequent reduction in brain parenchymal volume, ultimately leading to ventricular enlargement (26).

De Bonis, in one of his articles, observed a significant relationship between the superior limit of the craniectomy under 25 mm from the midline in patients who developed hydrocephalus (27). Likewise, in our series, out of 60% of the patients who developed hydrocephalus had undergone craniectomy, whose superior limit was ≤25 mm from the midline.

The evaluation of a craniectomy procedure necessitates a careful consideration of various adverse outcomes. The challenge lies in devising future research methodologies to explore the clinical effectiveness of the procedure. In this context, advanced outcome prediction models and extensive longitudinal cohort studies may be useful.

Our study is subject to various limitations. The study conducted was a retrospective, single-center investigation characterized by a somewhat limited sample size, which was indicative of the stringent criteria employed for inclusion and exclusion.

The preliminary determination to undertake the craniectomy was made based on the preoperative scan, but the ultimate decision was typically made once the condition of the brain and the cleared area following clot removal were observed. The unavailability of ICP data hindered our ability to conduct a comparative analysis between pre- and post-operative data, as well as to assess its correlation with craniectomy size or stratify it with pathology.

## In conclusion

In severe traumatic brain injury (TBI) necessitating decompressive craniectomy (DC), clinical outcomes are influenced less by surgical technique which is in general standardized, and more by factors such as the severity, type and extensions of the lesions, initial clinical status upon presentation, and possibly by post-operative intensive care management.

Our study suggests that a high percentage of patients who were on vasopressors and mechanical ventilation for a prolonged period developed hydrocephalus after DC. To our knowledge, this study is the first to show such a correlation. This highlights the importance of early extubation and minimizing vasopressor use to reduce the risk of PTH, taking into consideration the statistically significant correlation between the duration of mechanical ventilation and the development of postoperative hydrocephalus found in our data.

We are convinced that the continuation of this study and its multi-centre extension will allow us to find the right balance between the necessary measures of intensive care for patients with severe traumatic brain injury and the avoidance of complications generated by a possibly too-aggressive management.

Ultimately, the therapeutic goal in the case of severe head trauma patients is their survival, which is the first condition for neurological recovery.

## Data Availability

The raw data supporting the conclusions of this article will be made available by the authors, without undue reservation.
